# Elevated Periimplantation Uterine Natural Killer Cell Density in Human Endometrium Is Associated With Impaired Corticosteroid Signaling in Decidualizing Stromal Cells

**DOI:** 10.1210/jc.2013-1977

**Published:** 2013-09-11

**Authors:** Keiji Kuroda, Radha Venkatakrishnan, Sean James, Sandra Šućurović, Biserka Mulac-Jericevic, Emma S. Lucas, Satoru Takeda, Anatoly Shmygol, Jan J. Brosens, Siobhan Quenby

**Affiliations:** The Division of Reproductive Health (K.K., R.V., S.J., E.S.L., A.S., J.J.B., S.Q.), Clinical Science Research Laboratories, Warwick Medical School, Coventry CV2 2DX, United Kingdom; Department of Obstetrics and Gynaecology (K.K., S.T.), Juntendo University Faculty of Medicine, Tokyo 113-8421, Japan; and Department of Physiology and Immunology (S.Š., B.M.-J.), Medical School, University of Rijeka, Braće Branchetta 20, 51000 Rijeka, Croatia

## Abstract

**Background:**

Decidualizing human endometrial stromal cells (HESCs) profoundly up-regulate 11β-hydroxysteroid dehydrogenase type 1 (11βHSD1), the enzyme that converts inert cortisone to active cortisol. We postulated that the induction of a cortisol gradient upon decidualization of the periimplantation endometrium may impact on the uterine natural killer (uNK) cell population and on local expression of corticosteroid-dependent target genes.

**Methods:**

Midluteal endometrial biopsies (n = 55) were processed for uNK cell (CD56) analysis and primary HESC cultures. The cultures remained either untreated or were decidualized for 4 or 8 days. A tissue microarray was constructed from endometria with normal (n = 18) and elevated uNK cell (n = 18) scores. An abnormal uNK cell test was defined as greater than 5% CD56^+^ cells in the subluminal stroma.

**Results:**

Increased uNK cell density was associated with lower endometrial expression of 11βHSD1 and mineralocorticoid receptor (MR) but not glucocorticoid receptor in vivo. Elevated uNK cell density also corresponded to impaired induction of key decidual markers (11βHSD1, prolactin, and insulin-like growth factor binding protein-1) and MR-dependent enzymes (dehydrogenase/reductase member 3 and retinol saturase) in differentiating HESC cultures. Increased uNK cell density in vivo was not associated with increased in vitro expression of either IL-15 or IL-11, two cytokines implicated in uNK cell regulation.

**Conclusions:**

Elevated levels of uNK cells in the stroma underlying the surface epithelium are associated with inadequate cortisol biosynthesis by resident decidualizing cells and suboptimal induction of key MR-dependent enzymes involved in lipid biogenesis and the retinoid transport pathway. Our observations suggest that uNK cell testing identifies those women at risk of reproductive failure due to relative uterine cortisol deficiency.

Uterine natural killer (uNK) cells, an important component of the innate immune system, are the most abundant immune cells in midluteal (periimplantation) endometrium and in the decidua of early pregnancy (1). They represent a unique subset of natural killer cells, staining intensely for CD56 but not for CD16 antigens. uNK cells play a significant role in the establishment and maintenance of early pregnancy by promoting decidual angiogenesis, spiral artery remodeling, and trophoblast invasion (2, 3). In contrast to their circulating (CD56^+^/CD16^+^) counterparts, there is little evidence for a cytotoxic role of uNK cells at the fetomaternal interface. However, uNK cells express killer cell immunoglobulin-like receptors that preferentially bind to human leukocyte antigen-C molecules expressed on placental cells, suggesting a role in maternal allorecognition of fetal trophoblast (4). They are abundant around the spiral arteries, near endometrial glands, and adjacent to extravillous trophoblast in early pregnancy. Thus, uNK cells are unique in terms of their tissue distribution, phenotype, and function.

Both the maternal killer cell immunoglobulin-like receptor and fetal human leukocyte antigen-C gene systems are highly polymorphic and certain genotypic combinations are associated with a modest increase or decrease in pregnancy complications, including miscarriage, fetal growth restriction, and preeclampsia (4). In addition, several studies reported an association between elevated uNK cell levels in midluteal endometrium and reproductive failure (4–6). In particular, there is compelling evidence to link increased uNK density to recurrent pregnancy loss (RPL), defined here as three or more consecutive miscarriages. RPL is a prevalent disorder that affects 1%–2% of couples and a cause of considerable physical and psychological morbidity (7). Furthermore, RPL is associated with an increased likelihood of obstetric complications and adverse perinatal outcome in a subsequent ongoing pregnancy (8). Whether midluteal uNK cell testing in a nonconception cycle predicts subsequent pregnancy complications remains unresolved (9).

Resident human endometrial stromal cells (HESCs) are thought to serve as gatekeepers for the recruitment and distribution of immune cells in the periimplantation endometrium (10). For example, decidualizing (differentiating) HESCs secrete IL-11 and IL-15, two multifaceted cytokines implicated in trafficking and differentiation of uNK cells (11–13). uNK cells express the glucocorticoid receptor (GR) but lack progesterone receptor, rendering them directly responsive to cortisol but not progesterone (14, 15). Consistent with this notion, preconceptual glucocorticoid (prednisolone) treatment significantly reduces uNK cell density in RPL subjects as well as inhibiting endometrial angiogenesis (16, 17). We recently demonstrated that progesterone massively enhances the expression and activity of 11β-hydroxysteroid dehydrogenase type 1 (11βHSD1) in decidualizing HESCs (18), suggesting that local cortisol biosynthesis plays an integral role in the preparation of the endometrium for implantation. Decidualization is further associated with a decline in GR expression and reciprocal induction of the mineralocorticoid receptor (MR), which in turn drives the expression of several key enzymes involved in lipid and retinoid metabolism, including retinol saturase (RETSAT), various short-chain dehydrogenases/reductases (such as dehydrogenase/reductase (DHRS) member 3, DHRS4, and DHRS4L2), and steroidogenic acute regulatory protein-related lipid transfer protein 5 (18).

Emerging evidence suggests that aberrant differentiation of resident HESCs into specialized decidual cells is the hallmark of RPL (19, 20). Taken together, these observations raise the possibility that excessive uNK cell levels in midluteal endometrial samples reflect relative local corticosteroid deficiency, caused by inadequate induction of decidual 11βHSD1 and result in impaired local metabolic function.

## Materials and Methods

### Patient selection and endometrial sampling

The study was approved by the local ethics committee (1997/5065). Subjects were recruited in the Implantation Clinic, a dedicated research clinic at University Hospitals Coventry and Warwickshire National Health Service Trust for patients suffering RPL or recurrent in vitro fertilization treatment failure. Written informed consent was obtained prior to tissue collection. Endometrial biopsies were timed between 7 and 10 days after the preovulatory LH surge. Samples were obtained using a Wallach Endocell sampler (Wallach) under ultrasound guidance, starting from the uterine fundus and moving downward to the internal cervical ostium. Each biopsy was divided, with one part fixed in formalin for immunohistochemistry and the other processed for primary cell culture. The demographic details of participating subjects are summarized in Supplemental Tables 1 and 2, published on The Endocrine Society's Journals Online web site at http://jcem.endojournals.org.

### Primary cell culture

HESCs were isolated, cultured, and maintained as described (20). Primary cultures were passaged once, allowed to grow to confluency, and then decidualized with 0.5 mM 8-bromoadenosine cAMP (8-bromo-cAMP; Sigma), 1 μM progesterone (P4; Sigma), and 0.1 μM cortisone (E; Sigma). Cortisone, which is inactive, was added to decidualizing HESC cultures as the substrate for endogenous conversion by 11βHSD11 to cortisol (18).

### Immunohistochemistry

Five-micrometer-thick formalin-fixed, paraffin-embedded tissue sections were labeled with antibody to CD56 (NCL-CD56-1B6; Novacastra) using standard methods and detection systems (3). The uNK cell density was determined as the percentage of uNK cells within the stromal cell population. Because uNK cell density varies with endometrial depth, counting of CD56+ cells was confined to the stroma underlying the luminal epithelium. Five randomly selected high-power magnification fields per biopsy were assessed using ImageJ software (Rasband, W. S., ImageJ, National Institutes of Health) to minimize interobserver variability (21, 22). Normal uNK cell density was defined as 5% or less CD56^+^ cells in the stroma underlying the luminal epithelium (17, 23). A Mirax Midi slide scanner was used to scan bright-field sections with a ×20 objective with a resolution of 0.23 μm/pixel. This produces images that can be dynamically manipulated within the viewer software, allowing optical magnifications up to ×20 and digital magnification to ×200.

### Tissue microarray (TMA)

Areas of interest, ie, subepithelial regions, were spotted and tissue microarrays comprising duplicate 0.6-mm cores from 18 cases in each group were constructed using Alphelys TMA Designer R 2 version 1.0.0.8. Sections (3 μm) were cut from completed array blocks and transferred to silanized glass slides. Sections from these arrays were then stained for CD56, 1:200 (NCL-CD56-1B6; Novocastra); 11βHSD1, 1:300 (AB83522; Abcam); MR, 1:400 (H-300: SC-11412; Santa Cruz Biotechnology); and GR, 1:200 (E-20: SC-1003; Santa Cruz Biotechnology). Semiquantative analysis was performed using a Panoramic viewer to capture images. High-power (×400) images were analyzed with Image J (http://rsbweb.nih.gov/ij/) using the color deconvolution plugin and thresholding to assess the percentage of strongly immunopositive endometrial cells. The observers were blind as to the origin of the samples.

### Real-time quantitative PCR

Total RNA was extracted with RNA STAT-60 from primary HESC cultures. After treatment with amplification-grade deoxyribonuclease I (Invitrogen Ltd), cDNA was generated using the SuperScript II first-strand synthesis system for RT-PCR kit (Invitrogen). Template quantification was performed with an ABI Step One system (Applied Biosystems) using Power SYBR Green PCR master mix (Applied Biosystems). RNA input variances were normalized against the levels of the *L19* housekeeping gene, which encodes a ribosomal protein. All measurements were performed in duplicate. Specific primer pairs were designed using Primer3 software (http://frodo.wi.mit.edu): L19 sense, 5′-GCG GAA GGG TAC AGC CAA T-3′, L19-R antisense, 5′-GCA GCC GGC GCA AA-3′; 11βHSD1 sense, 5′-AGC AAG TTT GCT TTG GAT GG-3′, 11βHSD1 antisense, 5′-AGA GCT CCC CCT TTG ATG AT-3′; decidual prolactin (PRL) sense, 5′-AAG CTG TAG AGA TTG AGG AGC AAA C-3′, decidual PRL antisense, 5′-TCA GGA TGA ACC TGG CTG ACT A-3′; IGF-binding protein-1 (IGFBP1) sense, 5′-CGA AGG CTC TCC ATG TCA CCA-3′, IGFBP1 antisense, 5′-TGT CTC CTG TGC CTT GGC TAA AC-3′; IL-11 sense, 5′-CTC GAG TTT CCC CAG ACC CTC GG-3′, IL-11 antisense, 5′-TGT CAG CAC ACC TGG GAG CTG TAG-3′; IL-15 sense, 5′-TGG CTG CTG GAA ACC CCT TGC-3′, IL-15 antisense, 5′-CCC TGC ACT GAA ACA GCC CAA AA-3′; DHRS3 sense, 5′-AGC GCG GCG CCA GAA AGA TT-3′, DHRS3 antisense, 5′-TCA CCC ACC TTC TCC CGG ACG-3′; and RETSAT sense, 5′-CGC TGC CTG CCA GGT GTG AAG-3′, RETSAT antisense, 5′-AGA CGT AGC GCT CCA TCG CC-3′.

### Western blot analysis

Whole-cell protein extracts were obtained by direct lysis in Laemmli buffer heated to 100°C. Proteins resolved by SDS-PAGE were transferred to a polyvinyl difluoride membrane (GE Healthcare) and probed with antibodies raised against 11βHSD1, 1:1000 (AB83522; Abcam); DHRS3, 1:1000 (15393-1-AP; ProteinTech Group); RETSAT, 1:1000 (SAB1407586; Sigma); and β-actin, 1:100 000 (A1978; Sigma). After incubation with horseradish peroxidase-conjugated secondary antibodies diluted 1:2000 (DAKO), immunoreactivity was visualized using the ECL^+^ chemoluminescent detection kit (Amersham).

### Statistical analysis

Data were analyzed with the statistical package GraphPad Prism (GraphPad Software Inc). A Student's *t* test and a Mann-Whitney *U* test were used when appropriate. Logarithmic transformations were used when data were not normally distributed. Pearson's correlation coefficient (*r*) was used to assess the correlation between uNK cell densities in vivo and the induction of various genes upon decidualization of corresponding primary HESC cultures. Statistical significance was assumed when *P* < .05.

## Results

### Midluteal uNK cell density correlates inversely with endometrial 11βHSD1 expression

**Figure 1.**
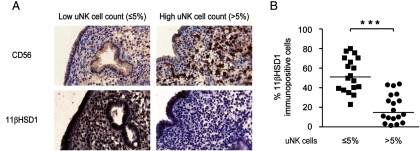
Inverse correlation between uNK cell density in the subluminal epithelium and expression of 11βHSD1. A, Representative CD56 and 11βHSD1 immunostaining in midluteal endometrial biopsies deemed to have normal or elevated uNK cell count (≤5% or >5% CD56^+^ cells in the stroma, respectively). Original magnification, ×20. B, Relative 11βHSD1 immunostaining after image analysis of a TMA containing biopsies with normal (≤5%; n = 18) or increased (>5%, n = 18) uNK cell density in the subluminal stroma. ***, *P* < .001.

We routinely assess uNK cell densities by CD56 immunostaining of timed (d LH+7 to LH+10) endometrial biopsies from women suffering reproductive failure. Based on previous studies, normal uNK cell density is defined as 5% or less CD56+ cells in the stroma underlying the luminal epithelium (17, 23). We speculated that an apparent excess of uNK cells in the periimplantation endometrium may reflect impaired 11βHSD1 expression and relative cortisol deficiency. To test this hypothesis, a TMA was constructed using biopsies with normal (n = 18) as well as elevated (n = 18) uNK cell levels. The TMA was stained with anti-CD56 and anti-11βHSD1 antibodies. As shown in Figure 1A, a strong inverse correlation was observed between uNK cell density and 11βHSD1 immunoreactivity. This negative correlation was confirmed by semiquantitative image analysis (Figure 1B).

### Elevated uNK cell density in vivo is associated with impaired induction of key decidual markers in vitro

**Figure 2.**
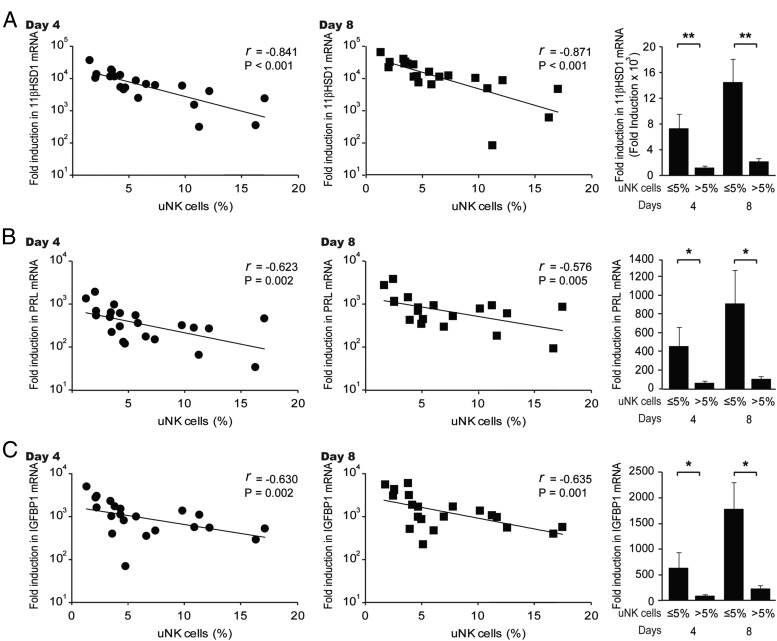
Inverse correlation between uNK cell densities in vivo and the induction of decidual markers in vitro. A, The uNK cell densities in midluteal biopsies correlated inversely to the induction of *11*β*HSD1* transcripts in primary HESCs decidualized for 4 days (left panel) or 8 days (middle panel). Note the logarithmic Y-axis. The right panel shows the mean (±SEM) induction of *11*β*HSD1* transcripts in biopsies deemed to have normal or elevated uNK cell counts. B, The uNK cell densities in midluteal biopsies correlated inversely to the induction of *PRL* transcripts in primary HESCs decidualized for 4 days (left panel) or 8 days (middle panel). The right panel shows the mean (±SEM) induction of *PRL* transcripts in biopsies deemed to have normal or elevated uNK cell counts. Note the logarithmic Y-axis. C, The uNK cell densities in midluteal biopsies correlated inversely to the induction of *IGFBP1* transcripts in primary HESCs decidualized for 4 days (left panel) or 8 days (middle panel). The right panel shows the mean (±SEM) induction of *PRL* transcripts in biopsies deemed to have normal or elevated uNK cell counts. Note the logarithmic Y-axis. *, *P* < .05; **, *P* < .01.

Previous studies have shown that an aberrant decidual response, associated with reproductive disorders such as endometriosis and RPL, is maintained in culture (24, 25). This prompted us to investigate whether uNK cell density in vivo could reflect the induction of 11βHSD1 in response to decidual cues. To this end, we divided timed endometrial biopsies and processed one part for CD56 immunostaining and the other part for primary HESCs. These cultures were passaged once, grown to confluency, and treated with 8-bromo-cAMP, P4, and E for either 4 or 8 days. As shown in Figure 2, there was a striking inverse correlation between uNK cell density in vivo and the responsiveness of paired primary cultures to differentiation stimuli (Supplemental Table 1). This inverse correlation was apparent for the induction of not only *HSD11B1*, the gene that encodes 11βHSD1 (Figure 2A) but also for *PRL* and *IGFBP1*, two classical decidual marker genes (Figure 2, B and C).

**Figure 3.**
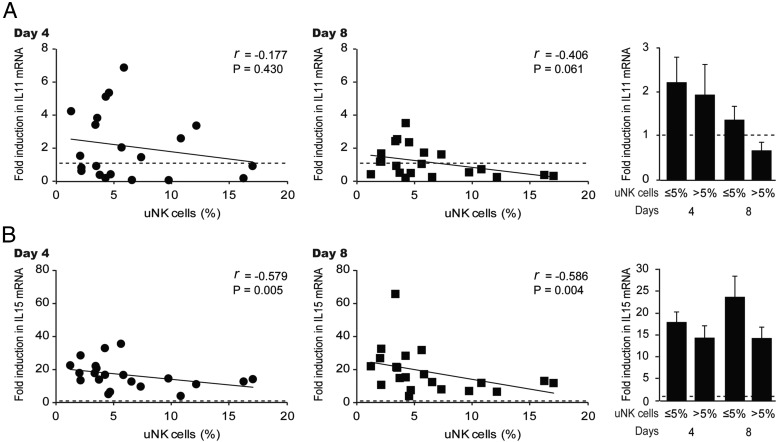
Induction of *IL11* and *IL15* mRNA in decidualizing HESCs in culture and uNK cell densities in vivo. A and B, The uNK cell densities in midluteal biopsies were correlated to the induction of *IL11* and *IL15* transcripts, respectively, in corresponding primary HESCs decidualized for either 4 days (left panel) or 8 days (middle panel). The right panel shows the mean (±SEM) induction in biopsies deemed to have normal or elevated uNK cell counts.

We also examined whether this association extended to IL-11 and IL-15, cytokines implicated in regulating uNK cells (11–13). Rather surprisingly, there was a significant trend toward higher levels of induction IL-15, but not IL-11, transcripts in decidualizing HESC cultures obtained from biopsies with normal uNK cell densities (Figure 3, A and B).

### Elevated uNK cell density in vivo is associated with impaired expression of MR-dependent metabolic enzymes

**Figure 4.**
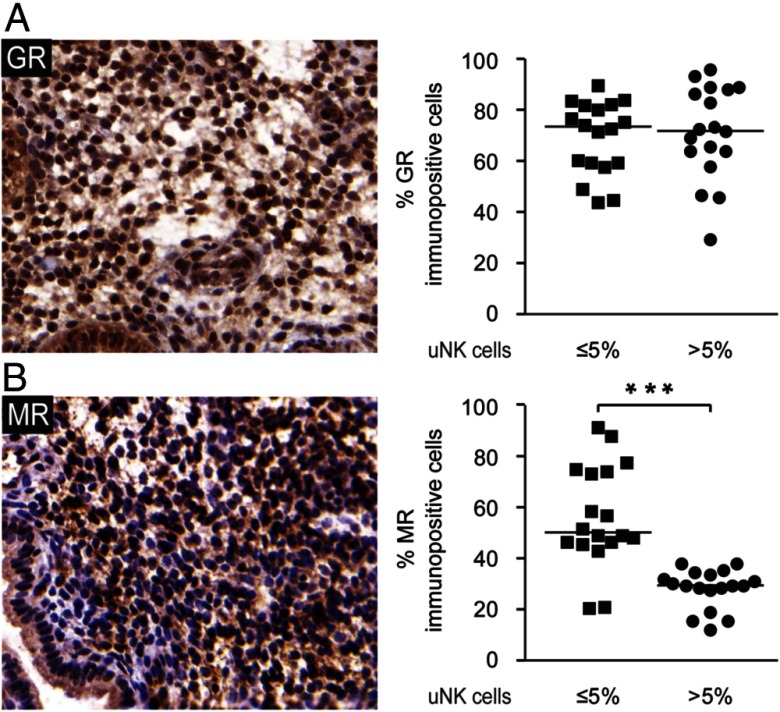
Increased uNK cell levels are associated with impaired MR expression. A and B, The expression of GR and MR, respectively, in the subluminal stroma was assessed by immunostaining of a TMA containing biopsies with normal (≤5%; n = 18) or increased (>5%, n = 18) uNK cell density in the subluminal stroma. The left panel shows representative immunostaining (original magnification, ×40), whereas the right panel depicts semiquantitative image analysis of the immune staining. ***, *P* < .001.

Next, we used the TMA to examine the expression of the cortisol-responsive receptors, GR and MR. As reported by others (26), GR and MR are both expressed in the endometrial stroma (Figure 4A). Semiquantitative analysis of the TMA showed no difference in stromal GR immunoreactivity in biopsies characterized by elevated vs normal uNK cell levels. In contrast, an increased percentage of CD56^+^ cell density was associated with significantly lower MR expression levels (*P* < .05; Figure 4B).

**Figure 5.**
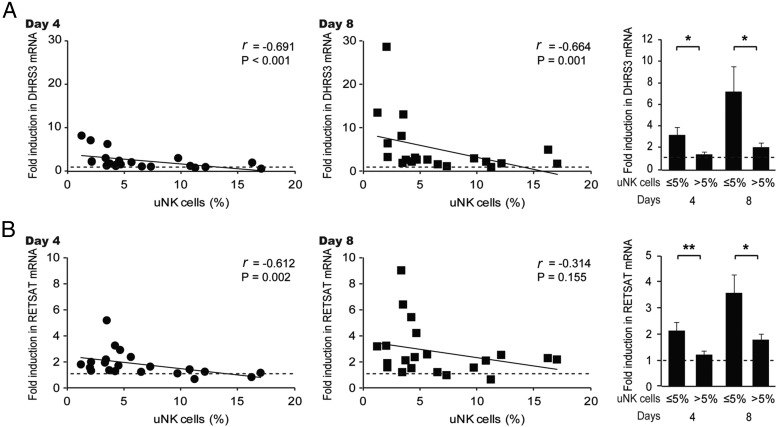
Elevated uNK cell density is associated with blunted expression of the MR-dependent genes in decidualizing HESCs. A, The uNK cell densities in 21 biopsies correlated inversely to the induction of *DHRS3* transcripts in primary HESCs decidualized for 4 days (left panel) or 8 days (middle panel). The right panel shows the mean (±SEM) induction of *DHRS3* transcripts in biopsies deemed to have normal or elevated uNK cell counts. B, The uNK cell densities in 21 biopsies correlated inversely to the induction of *RETSAT* transcripts in primary HESCs decidualized for 4 days (left panel) or 8 days (middle panel). The right panel shows the mean (±SEM) induction of *RETSAT* transcripts in biopsies deemed to have normal or elevated uNK cell counts. *, *P* < .05; **, *P* < .01.

Previous knockdown experiments in decidualizing HESCs have shown that the 11βHSD1/MR axis regulates the expression of several enzymes involved in lipid metabolism and retinoid acid biosynthesis and storage (18). We measured the expression levels of two target genes, *DHRS3* and *RETSAT*, to monitor the MR activity in decidualizing primary HESC cultures established from biopsies with normal or elevated uNK cell levels. Both genes were moderately induced upon treatment with 8-bromo-cAMP/P4/E in a time-dependent manner when the primary HESC cultures were established from samples with 5% or less CD56+ cells in the subepithelial stroma (Figure 5). By contrast, this induction was significantly impaired in cultures established from high uNK cell samples.

**Figure 6.**
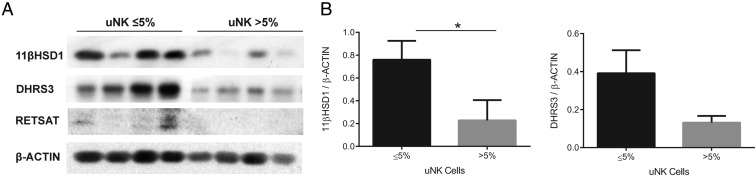
A, Composite figure showing 11βHSD1, DHRS3, and RETSAT protein expression in primary HESC cultures decidualized for 4 days. A total of eight primary cultures were established from biopsies with normal or elevated uNK cell densities. β-Actin served as a loading control. B, Semiquantitative analysis of 11βHSD1 and DHRS3 expression relative to β-actin. Because of the low level of expression, RETSAT expression was not quantified. *, *P* < .05.

To validate these findings, we performed Western blot analysis of primary HESCs decidualized with 8-bromo-cAMP/P4/E for 4 days. As shown in Figure 6A, 11βHSD1 and DHRS3 were abundantly expressed in primary cultures decidualized for 4 days. Induction of RETSAT, however, requires prolonged decidualization. This enzyme was barely detectable after 4 days of differentiation and only in primary cultures established from samples with 5% or less CD56+ cells in the subepithelial stroma. Semiquantitative analysis of the blots showed that elevated uNK cell density in vivo is associated with significantly lower 11βHSD1 expression in corresponding decidualizing primary HESC cultures and a trend toward lower DHRS3 levels (*P* = .03 and *P* = .07, respectively; Figure 6B).

## Discussion

Increased uNK cell density in midluteal endometrium has been associated with reproductive failure, especially RPL (4–6) However, the mechanisms that account for cyclic recruitment of uNK cell precursors and subsequent proliferation and differentiation within the periimplantation environment are not well understood. In addition to IL-11 and IL-15, several other endometrial factors may be implicated in this process, including chemokine motif ligand 14, IL-12, and IL-33 (25, 27, 28). Different strands of evidence suggest that induction of a cortisol gradient upon decidualization of HESCs is also a key regulator of uNK cells in periimplantation endometrium. First, preconceptual prednisolone treatment markedly reduces uNK cell density in RPL patients (17). uNK cells express GR but not progesterone receptors (15), the inference being that glucocorticoids are likely to act directly on these cells. Second, we found a strong negative correlation between uNK cell densities and expression of 11βHSD1 in differentiating stromal cells in vivo. We have shown previously that 11βHSD1 expression and enzyme activity in decidualizing HESCs is driven by cAMP and P4 signaling (18). Furthermore, inhibition of 11βHSD1 activity with either carbenoxolone disodium salt or PF 915275 virtually abolishes the induction of *HSD11B1*, indicating that local cortisol signaling reinforces the expression of this enzyme in decidualizing cells through an autocrine mechanism (18). By contrast, the expression of the type 2 isoform (11βHSD2), the dehydrogenase that converts cortisol into inactive cortisone, is low in both undifferentiated and decidualizing HESCs (18). Notably, the decidual process in the human endometrium is under tight spatiotemporal control (29). It is initiated in the midluteal phase of the cycle first in stromal cells surrounding the terminal spiral arteries and underlying the luminal epithelium. Thus, rather than the total tissue concentration of uNK cells, it is possible that excessive migration of uNK cells from their usual position in the basal and perivascular regions of the endometrium to the subluminal region is the hallmark of an abnormal decidual response that predisposes for early pregnancy loss. Finally, there is increasing evidence that the responsiveness of endometrial cells to differentiation signals is subject to epigenetic programming (30), which explains how an aberrant decidual response in vivo is maintained, at least partly, upon differentiation of purified HESCs in vitro (24, 25, 31). In agreement, we found that high uNK cell density in vivo is associated with blunted induction of *HSD11B1* as well as decidual marker genes, such as *PRL* and *IGFBP1*, in primary cultures. This strong inverse correlation suggests that the 5% threshold of uNK cell density is somewhat arbitrary. Whether increased uNK cells densities correlate to increased risk of pregnancy failure warrants further investigation.

In addition to decidual marker genes, induction of IL-11 and IL-15 also tended to be lower in decidualizing cells established from biopsies with elevated uNK cell levels. This observation does not exclude that expression levels of these cytokines in situ correlate with uNK cell levels as reported for IL-15 in a recent study (32).

Human uNK cells have been described as immature and inactive before pregnancy (1). How elevated levels of uNK cells prior to conception predispose to subsequent pregnancy failure is unclear. Ablation of these cells in mice has been shown to compromise spiral arteriole remodeling and maintenance of decidual integrity seen after midpregnancy (3, 33). Yet uNK cell-deficient IL-15^−/−^ mice are fertile and have normal gestation lengths and litter sizes comparable with wild-type mice (34). Similarly, human uNK cells are implicated in spiral artery remodeling. They are a rich source of angiogenic growth factors, although paradoxically the endometrium of RPL patients is characterized by reduced expression of several key factors, including platelet-derived growth factor-BB, angiotensin-2, vascular endothelial growth factor-A, and vascular endothelial growth factor-C (35). Our data suggest that increased density of CD56^+^ cells in the subluminal endometrial stromal compartment may be an indirect marker of local corticosteroid deficiency. Furthermore, our data indicate that the 11βHSD1/MR axis in target cells may be particularly affected as exemplified by the impaired induction of *DHRS3* and *RETSAT* transcripts in decidualizing cultures established from high uNK cell biopsies. These enzymes are involved in lipid metabolism and retinoid acid (RA) biosynthesis and storage. Both shortage and excess of RA contribute to fetal malformation, suggesting that retinoid metabolism must be regulated closely at the fetomaternal interface (36). Alcohol dehydrogenase and nicotinamide adenine dinucleotide phosphate oxidase-dependent short-chain dehydrogenases/reductases, including DHRS3, oxidize retinal to retinol and promote its storage as retinyl esters. Likewise, RETSAT is involved in the regulation of retinoid storage as lipid droplets (37). Interestingly, RA inhibits decidualization of HESCs, and excess levels of RA or retinal are cytotoxic (38). Thus, high uNK cell density in the periimplantation endometrium may be associated with perturbations in the retinoid metabolism pathway in the stromal compartment, which in turn predisposes for an impaired decidual response and compromise histiotrophic support of the early conceptus.

Thus, it seems likely that complex and dynamic gradients of chemoattractants and chemorepellents control the spatiotemporal distribution of uNK cells in the periimplantation endometrium. In addition to glands and other immune cells, decidualizing stromal cells play a major role in governing this process, at least partially, by inducing a cortisol gradient that establishes a nutritive environment essential for postimplantation embryo development and fetal growth. Our data suggest that excessive uNK cells in the subluminal stromal compartment prior to conception may serve as a potential biomarker for a suboptimal decidual response in pregnancy. A recent pilot randomized, double-blind controlled clinical trial suggested an improvement in live birth rate with prednisolone in women with RPL and high midluteal uNK cell density (23), although this finding needs validating in a larger trial. In addition, assessment of uNK cell density varies greatly from laboratory to laboratory and intercycle variation has been reported (22). To be clinically useful, international standardization of uNK cell assessment is urgently needed.

## References

[1] Manaster I Mizrahi S Goldman-Wohl D. Endometrial NK cells are special immature cells that await pregnancy. J Immunol. 2008;181:1869–1876 18641324 10.4049/jimmunol.181.3.1869

[2] Hanna J Goldman-Wohl D Hamani Y. Decidual NK cells regulate key developmental processes at the human fetal-maternal interface. Nat Med. 2006;12(9):1065–74 16892062 10.1038/nm1452

[3] Lash GE Robson SC Bulmer JN Review: functional role of uterine natural killer (uNK) cells in human early pregnancy decidua. Placenta. 2010;31(Suppl):S87–S92 20061017 10.1016/j.placenta.2009.12.022

[4] Chazara O Xiong S Moffett A. Maternal KIR and fetal HLA-C: a fine balance. J Leukoc Biol. 2011;90:703–716 21873457 10.1189/jlb.0511227

[5] Quenby S Nik H Innes B Lash G Turner M Drury J Bulmer J. Uterine natural killer cells and angiogenesis in recurrent reproductive failure. Hum Reprod. 2009;24:45–54 18835875 10.1093/humrep/den348

[6] Clifford K Flanagan AM Regan L. Endometrial CD56+ natural killer cells in women with recurrent miscarriage: a histomorphometric study. Hum Reprod. 1999;14:2727–2730 10548610 10.1093/humrep/14.11.2727

[7] Rai R Regan L. Recurrent miscarriage. Lancet. 2006;368:601–611 16905025 10.1016/S0140-6736(06)69204-0

[8] Jauniaux E Farquharson RG Christiansen OB Exalto N. Evidence-based guidelines for the investigation and medical treatment of recurrent miscarriage. Hum Reprod. 2006;21:2216–2222 16707507 10.1093/humrep/del150

[9] Tang AW Alfirevic Z Turner MA Drury JA Small R Quenby S. A feasibility trial of screening women with idiopathic recurrent miscarriage for high uterine natural killer cell density and randomising to prednisolone or placebo when pregnant. Hum Reprod. 2013;28(7):1743–1752 23585559 10.1093/humrep/det117

[10] Nancy P Tagliani E Tay CS Asp P Levy DE Erlebacher A. Chemokine gene silencing in decidual stromal cells limits T cell access to the maternal-fetal interface. Science. 2012;336:1317–1321 22679098 10.1126/science.1220030 PMC3727649

[11] Ain R Trinh ML Soares MJ. Interleukin-11 signaling is required for the differentiation of natural killer cells at the maternal-fetal interface. Dev Dyn. 2004;231:700–708 15499555 10.1002/dvdy.20183

[12] Ashkar AA Black GP Wei QX. Assessment of requirements for IL-15 and IFN regulatory factors in uterine NK cell differentiation and function during pregnancy. J Immunol. 2003;171:2937–2944 12960317 10.4049/jimmunol.171.6.2937

[13] Godbole G Modi D. Regulation of decidualization, interleukin-11 and interleukin-15 by homeobox A 10 in endometrial stromal cells. J Reprod Immunol. 2010;85:130–139 20478621 10.1016/j.jri.2010.03.003

[14] Henderson TA Saunders PTK Moffett-King A Groome NP Critchley HOD. Steroid receptor expression in uterine natural killer cells. J Clin Endocrinol Metab. 2003;88:440–449 12519888 10.1210/jc.2002-021174

[15] Guo W Li PF Zhao GF Fan HY Hu YL Hou YY. Glucocorticoid receptor mediates the effect of progesterone on uterine natural killer cells. Am J Reprod Immunol. 2012;67:463–473 22380541 10.1111/j.1600-0897.2012.01114.x

[16] Lash GE Bulmer JN Innes BA Drury JA Robson SC Quenby S. Prednisolone treatment reduces endometrial spiral artery development in women with recurrent miscarriage. Angiogenesis. 2011;14:523–532 21984529 10.1007/s10456-011-9237-x

[17] Quenby S Kalumbi C Bates M Farquharson R Vince G. Prednisolone reduces preconceptual endometrial natural killer cells in women with recurrent miscarriage. Fertil Steril. 2005;84:980–984 16213853 10.1016/j.fertnstert.2005.05.012

[18] Kuroda K Venkatakrishnan R Salker SM. Induction of 11β-hydroxysteroid dehydrogenase type 1 and activation of distinct mineralocorticoid receptor- and glucocorticoid receptor-dependent gene networks in decidualizing human endometrial stromal cells. Mol Endocrinol. 2013;27(2):192–202 23275455 10.1210/me.2012-1247 PMC5417328

[19] Salker M Teklenburg G Molokhia M. Natural selection of human embryos: impaired decidualization of the endometrium disables embryo-maternal interactieons and causes recurrent pregnant loss. PLoS One. 2010;7(12):e52252 20422017 10.1371/journal.pone.0010287 PMC2858209

[20] Salker SM Christian M Steel JH. Deregulation of the serum- and glucocorticoid-inducible kinase SGK1 in the endometrium causes reproductive failure. Nat Med. 2011;17(11):1509–1513 22001908 10.1038/nm.2498

[21] Drury JA Nik H van Oppenraaij RHF Tang A-W Turner MA Quenby S. Endometrial cell counts in recurrent miscarriage: a comparison of counting methods. Histopathology 2011;59(6):1156–1162 22175895 10.1111/j.1365-2559.2011.04046.x

[22] Mariee N Tuckerman E Ali A Li W Laird S Li TC. The observer and cycle-to-cycle variability in the measurement of uterine natural killer cells by immunohistochemistry. J Reprod Immunol. 2012;95:93–100 22906826 10.1016/j.jri.2012.05.001

[23] Tang AW Alfirevic Z Turner MA Drury JA Small R Quenby S. A feasibility trial of screening women with idiopathic recurrent miscarriage for high uterine natural killer cell density and randomizing to prednisolone or placebo when pregnant. Hum Reprod. 2013;28:1743–1752 23585559 10.1093/humrep/det117

[24] Klemmt PA Carver JG Kennedy SH Koninckx PR Mardon HJ. Stromal cells from endometriotic lesions and endometrium from women with endometriosis have reduced decidualization capacity. Fertil Steril. 2006;85:564–572 16500320 10.1016/j.fertnstert.2005.08.046 PMC1626574

[25] Salker MS Nautiyal J Steel JH. Disordered IL-33/ST2 activation in decidualizing stromal cells prolongs uterine receptivity in women with recurrent pregnancy loss. PLoS One. 2012;7:e52252 23300625 10.1371/journal.pone.0052252 PMC3531406

[26] McDonald SE Henderson TA Gomez-Sanchez CE Critchley HO Mason JI. 11β-Hydroxysteroid dehydrogenases in human endometrium. Mol Cell Endocrinol. 2006;248:72–78 16406280 10.1016/j.mce.2005.12.010

[27] Karsten CM Behrends J Wagner AK. DC within the pregnant mouse uterus influence growth and functional properties of uterine NK cells. Eur J Immunol. 2009;39:2203–2214 19593769 10.1002/eji.200838844

[28] Mokhtar NM Cheng CW Cook E Bielby H Smith SK Charnock-Jones DS. Progestin regulates chemokine (C-X-C motif) ligand 14 transcript level in human endometrium. Mol Hum Reprod. 2010;16:170–177 19903701 10.1093/molehr/gap100

[29] Gellersen B Brosens IA Brosens JJ. Decidualization of the human endometrium: Mechanisms, functions, and clinical perspectives. Semin Reprod Med. 2007;25:445–453 17960529 10.1055/s-2007-991042

[30] Grimaldi G Christian M Steel JH Henriet P Poutanen M Brosens JJ. Down-regulation of the histone methyltransferase EZH2 contributes to the epigenetic programming of decidualizing human endometrial stromal cells. Mol Endocrinol. 2011;25:1892–1903 21903722 10.1210/me.2011-1139 PMC3198959

[31] Aghajanova L Horcajadas JA Weeks JL. The protein kinase A pathway-regulated transcriptome of endometrial stromal fibroblasts reveals compromised differentiation and persistent proliferative potential in endometriosis. Endocrinology. 2010;151:1341–1355 20068008 10.1210/en.2009-0923 PMC2840687

[32] Mariee N Li TC Laird SM. Expression of leukaemia inhibitory factor and interleukin 15 in endometrium of women with recurrent implantation failure after IVF; correlation with the number of endometrial natural killer cells. Hum Reprod. 2012;27:1946–1954 22537815 10.1093/humrep/des134

[33] Croy BA He H Esadeg S. Uterine natural killer cells: insights into their cellular and molecular biology from mouse modelling. Reproduction (Cambridge, England). 2003;126:149–160 10.1530/rep.0.1260149 PMC2967520 12887272

[34] Barber EM Pollard JW. The uterine NK cell population requires IL-15 but these cells are not required for pregnancy nor the resolution of a *Listeria monocytogenes* infection. J Immunol. 2003;171:37–46 12816981 10.4049/jimmunol.171.1.37

[35] Lash GE Innes BA Drury JA Robson SC Quenby S Bulmer JN. Localization of angiogenic growth factors and their receptors in the human endometrium throughout the menstrual cycle and in recurrent miscarriage. Hum Reprod. 2012;27:183–195 22081249 10.1093/humrep/der376

[36] Han BC Xia HF Sun J Yang Y Peng JP. Retinoic acid-metabolizing enzyme cytochrome P450 26a1 (cyp26a1) is essential for implantation: functional study of its role in early pregnancy. J Cell Physiol. 2010;223:471–479 20112286 10.1002/jcp.22056

[37] Schupp M Lefterova MI Janke J. Retinol saturase promotes adipogenesis and is downregulated in obesity. Proc Nat Acad Sci USA. 2009;106:1105–1110 19139408 10.1073/pnas.0812065106 PMC2633572

[38] Brar AK Kessler CA Meyer AJ Cedars MI Jikihara H. Retinoic acid suppresses in vitro decidualization of human endometrial stromal cells. Mol Hum Reprod. 1996;2:185–193 9238678 10.1093/molehr/2.3.185

